# Editorial: Substance-based medical devices for human health: a challenge of efficacy, safety, and sustainability

**DOI:** 10.3389/fdsfr.2023.1222790

**Published:** 2023-05-31

**Authors:** Alessandro Mugelli, Juan Tamargo

**Affiliations:** ^1^ Department of NEUROFARBA, University of Florence, Florence, Italy; ^2^ Department of Pharmacology and Toxicology, School of Medicine, Complutense University of Madrid, Spain

**Keywords:** substance-based medical device, efficacy and safety, European regulation, therapeutic effect, innovation

Alessandro Mugelli and Juan Tamargo Treatments without scientific proof of efficacy and safety are offered to and often used by individuals for their medical needs.

Substance-based medical devices (SBMDs) are medical devices composed of substances or by combinations of substances as defined in Annex VIII, Rule 21 of the European Medical Device Regulation (MDR) (https://eur-lex.europa.eu/legal-content/EN/TXT/?uri=CELEX%3A32017R0745). While SBMDs are similar to medicinal products (MPs) in their presentation and pharmaceutical form, they should achieve their therapeutic effect via a “*non*-*pharmacological*, *immunological*, *or metabolic mechanism of action.*”

Independently of the mechanism of action to reach their therapeutic effect, what is of utmost importance is that the therapeutic claim must be demonstrated by well-designed clinical trials and that the benefit/risk ratio reported when SBMDs are marketed should be corroborated by active postmarketing surveillance. Manufacturers must verify the quality and safety of the substances that are in the SBMD according to the EU legislation for patient safety, but this field also represents an opportunity for innovation and research.

The new MDR has been in force since May 2021. As clearly reported in the perspective articles by Giovagnoni, “the MDR’s inclusion of different types of product has created a significant opportunity for innovation.” In fact, “the Regulation allowed to repurpose the therapeutic properties of natural complex substances, which were unused, or even considered complementary and alternative medicine, within an evidence-based framework and as part of the healthcare sector.”

The aim of this Research Topic is to give the clear message that the healthcare system, the scientific research community, and the industry should be prepared to accept the challenges of this profound regulatory change, transforming this change into opportunities for innovation and health improvement.

Interestingly, in the EU, the regulatory changes on clinical research of MPs as well as the more general regulatory framework of medical devices, and in particular of SBMDs, have undergone a major parallel revision, in the interest of the wellbeing of the citizens, of the quality of science, and of improving study feasibility and homogeneity among EU nations (Rasi and Mugelli). This is particularly important because the SBMD market is increasing and currently represents 11% of the total self-medication market in the EU (Bilia et al., 2021).

SBMDs largely fulfill common medical needs in conditions where drugs can be safely and effectively replaced: this is clearly shown by some examples reported in the Research Topic. For example, in the long-term treatment of obese children and adolescents, an SBMD made of natural fiber complexes has been used in combination with a low-glycemic index diet with or without metformin and reduced body mass index and waist circumference, improved insulin sensitivity with reduction of glucose-metabolism abnormalities, increased insulin reserves, and, finally, improved circulating lipid profiles have been noted (Guarino. et al.; Stagi).

Further medical conditions where SBMDs are used with good efficacy and safety are various gastroenterological functional illnesses where pharmacological agents have limitations. Examples are functional esophageal disorders with typical symptoms (mainly heartburn and regurgitation) not associated with structural, inflammatory, or major motility abnormalities and functional dyspepsia characterized by symptoms like post-prandial fullness, early satiation, epigastric pain, and epigastric burning. Furthermore, several chronic gastrointestinal disorders have no underlying anatomic abnormalities identifiable by routine diagnostic examinations and are characterized by predominant symptoms of abdominal pain, bloating, distension, and/or bowel abnormalities (constipation, diarrhea, or mixed constipation and diarrhea), as in the case of irritable bowel syndrome, functional constipation, and functional diarrhea (Longstreth et al., 2006). The rationale for using SBMDs in these rather common conditions and the randomized and controlled clinical trials able to confirm their efficacy and safety are reviewed by Savarino V et al.

Overall, it is apparent that the MDR requires a demonstration of the claim of clinical efficacy and safety of an SBMD, and, consequently, it is also of paramount relevance to develop studies to investigate their non-pharmacological mechanisms of action. An example of an *in vitro* mechanistic study is reported by Bassetto et al., with the aim of providing scientifically validated procedures that may contribute to the definition of standard methods assessing the biological evaluation of SBMDs.

Finally, Marchesi et al. discuss the difficulties of classifying some products “neatly and unambiguously as belonging to a regulatory class” (MP, SBMD, food supplement, or food for special medical purposes). They use the case of citicoline in glaucoma because several criteria (commercial strategy, ease of market access, price/reimbursement, and mechanism of action) influence the final classification. Glaucoma is a relevant clinical problem; it affects 67 million people worldwide and is the second leading cause of irreversible blindness. Drugs commonly used for glaucoma aim to decrease intraocular pressure and are mostly administered as eye drops. Complementary and alternative medicine is used as adjuncts to traditional drugs (i.e., prostaglandin analogs, beta-blockers, alpha agonists, carbonic anhydrase inhibitors, and rho kinase inhibitors); oral food supplements and SBMDs (usually as eye drops) are widely used. It is estimated that 5%–15% of glaucoma patients take some form of alternative medicine based only on their impression that it can treat their glaucoma (Hetherington, 2013). In their article, the authors consider citicoline as a significant case study because citicoline has been used as a drug, food supplement, food for special medical purposes, and can be sold as an SBMD. They discuss how difficult it could be to discriminate between a pharmacological and a non-pharmacological mode of action because the mode of action may differ according to associated variables, such as the route of administration, dose, and selection of one of many possible targets.

**FIGURE 1:**
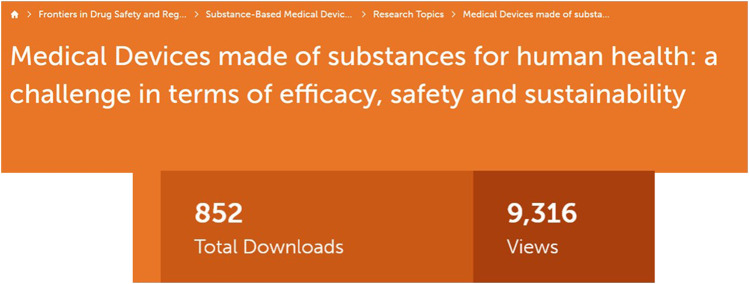
Total Downloads and Views of the Research Topic, accessed on May 29, 2023.

SBMDs may create a significant opportunity to promote innovation in patient management, increasing the spectrum of treatment choices with a favorable benefit/risk ratio. The close collaboration of pharmacologists, clinicians, and regulators is absolutely necessary to fully exploit this potential for growth. With this Research Topic, we hope to give the reader an overview of this new and unknown field that will likely have a relevant impact on the future of population health. The readers’ interest, as shown in Figure 1, appears to support the relevance of this evolving field.
